# Risk of Sleep Disorders among Patients with Tourette Syndrome: A Population-Based Cohort Study in Taiwan

**DOI:** 10.7150/ijms.107983

**Published:** 2025-04-22

**Authors:** Ning-Jen Chung, Yung-Rung Lai, Yih Yang, Shuo-Yan Gau, Shiang-Wen Huang, Tung-Han Tsai, Kuang-Hua Huang, Chien-Ying Lee

**Affiliations:** 1 School of Medicine, Chung Shan Medical University, Taichung 40201, Taiwan.; 2 Department of Pharmacology, Chung Shan Medical University, Taichung 40201, Taiwan.; 3 Department of Pharmacy, Chung Shan Medical University Hospital, Taichung 40201, Taiwan.; 4 Department of Surgery, E-Da Hospital, I-Shou University, Kaohsiung 824, Taiwan.; 5 Department and Graduate Institute of Business Administration, National Taiwan University, Taipei 106, Taiwan.; 6 Department of Health Services Administration, China Medical University, Taichung 406040, Taiwan.

**Keywords:** Tourette syndrome, sleep disorders, children, real-world evidence

## Abstract

**Background:** Tourette syndrome (TS) is a complex neurodevelopmental disorder often linked with various neuropsychiatric comorbidities. This population-based cohort study examined the association between TS and sleep disorders.

**Materials and methods:** Utilizing data from a nationwide database, this retrospective cohort study assessed the risk of sleep disorders in patients with TS. We enrolled 13,646 patients with new-onset TS from 2002 to 2015, each matched with four controls by age, sex, insured salary, urbanization level, Charlson comorbidity index, and year of inclusion. Follow-up continued until the development of sleep disorders, death, or the end of 2018. The risk was evaluated using a Cox proportional hazards model, with sensitivity analyses at ≤1, 1-5, and >5 years.

**Results:** After adjusting for several variables, patients with TS had a higher risk of sleep disorders (adjusted hazard ratio [aHR] = 1.76, 95% confidence interval [CI] = 1.58-1.96). Those aged over 18 years had a higher risk than those under 7 years (aHR = 7.76, 95% CI = 6.32-9.53). Patients with comorbid attention deficit hyperactivity disorder (ADHD) and anxiety also showed increased risks (aHR = 1.35, 95% CI = 1.09-1.67 and aHR = 2.33, 95% CI = 1.88-2.88, respectively). Sensitivity analyses confirmed a higher risk of sleep disorders in TS patients at <1-, 1-5-, and >5-year follow-up periods.

**Conclusion:** TS is a significant risk factor for sleep disorders. Patients with comorbid ADHD and anxiety are particularly at higher risk for sleep disturbances.

## Introduction

Tic disorders (TDs) are a class of neuropsychiatric disorders that develop in childhood and adolescence (before the age of 18 years); TDs are characterized by involuntary motor tics and vocal tics, and Tourette syndrome (TS) is the most severe form of TD 1, 2. TS is characterized by fluctuating *motor* tics and vocal* tics* 3. Epidemiological studies have revealed that motor tics *typically begin* at the age of 4-6 *years*, and the mean *age* at onset is 6 *years* 4, 5.

Patients with TS exhibit significantly decreased sleep quality as well as difficulties initiating and maintaining sleep 6. Patients with TS often present with several comorbidities. Among these comorbidities, attention deficit hyperactivity disorder (ADHD) is the most common comorbidity, followed by obsessive compulsive disorder (OCD), with prevalence rates of 50%-60% and 36%-50%, respectively 1, 3.

Little knowledge is available regarding the risk of sleep disorder in patients with TS; a nationwide database-based epidemiological study should be conducted to investigate the risk of sleep disorders among patients with TS. Patients with TS comorbid with neuropsychiatric disorders may have an increased likelihood of sleep problems**.** In this study, by using data from the National Health Insurance Research Database (NHIRD) in Taiwan, we investigated neuropsychiatric comorbidities as risk factors for the risk of incident sleep disorders among patients with TS.

## Materials and methods

### Data source

This study conducted a secondary analysis of 2001-2018 NHIRD data. The NHIRD is a nationally representative database maintained by the Health and Welfare Data Science Center (HWDC) of the Ministry of Health and Welfare, Taiwan, and contains the detailed clinical records and the inpatient and outpatient claims of the beneficiaries of Taiwan's National Health Insurance (NHI) program. The HWDC provides scrambled random identification numbers for insured patients to ensure their privacy. The NHI program has provided coverage for up to 99% of the country's population since 1995. The NHIRD provides real-world evidence to support clinical decisions and health-care policy-making 7, 8. Diagnostic data in the NHIRD before 2016 and after 2016 are, respectively, coded using International Classification of Diseases, Ninth Revision, Clinical Modification (ICD-9-CM) and International Classification of Diseases, Tenth Revision, Clinical Modification (ICD-10-CM) codes.

### Ethics statements

This study was conducted in accordance with the Declaration of Helsinki. The data used in the present study are anonymous to ensure the privacy of beneficiaries. Informed consent from participants was not required given that the database in this study contains only de-identified data. The present study was reported in accordance with the STROCSS criteria 9. The study protocol received ethical review and approval by the Institutional Review Board of Chung Shan Medical University Hospital, Taiwan (No. CSMUH CS1-22174).

### Study participants

**Figure 1:**
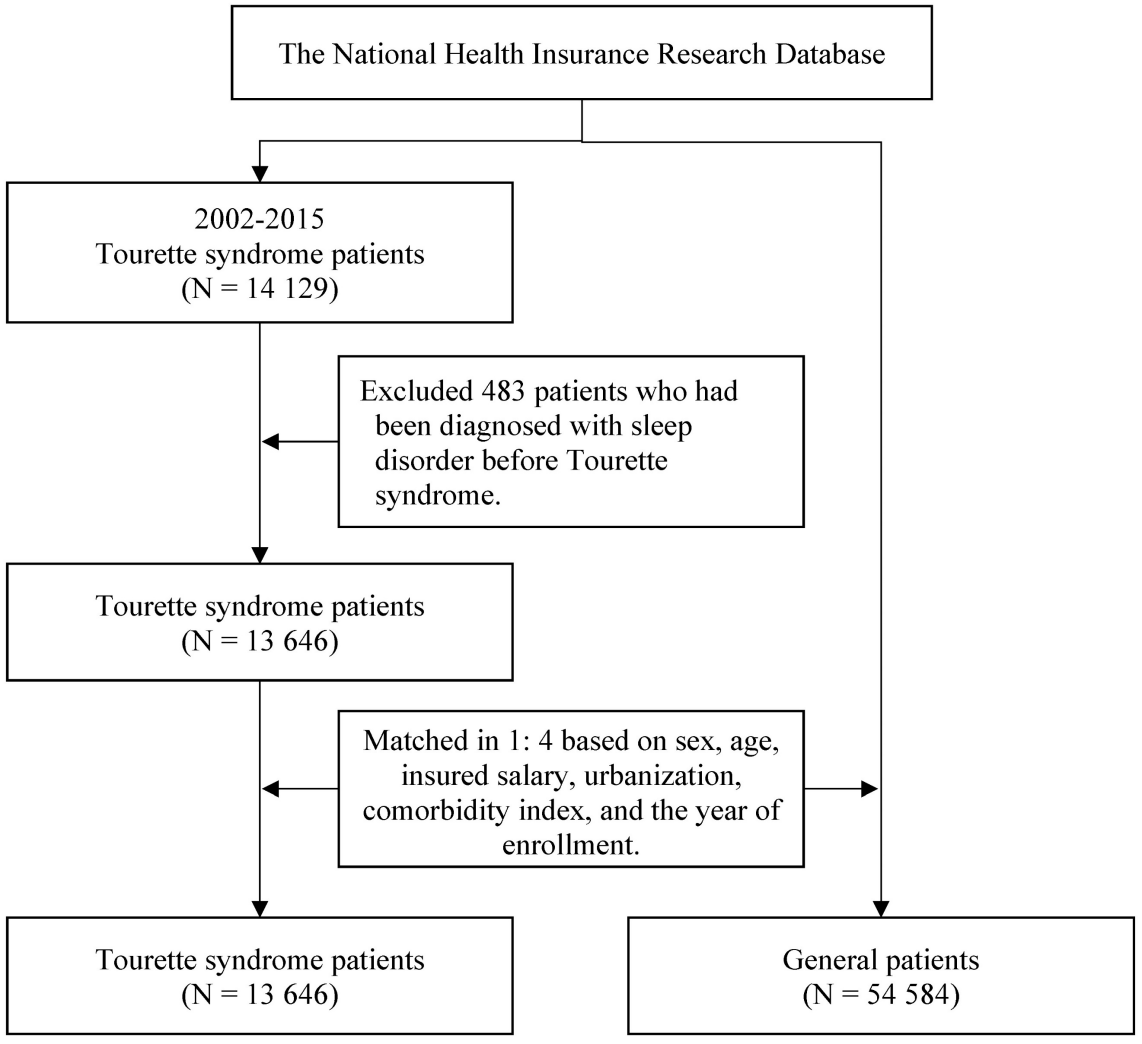
The flowchart of participant selection.

In this study, we enrolled patients with new-onset TS between 2002 and 2015. TS (ICD-9-CM code 307.23) was defined by the presence of three or more outpatient records of TS diagnoses in 1 year. Patients were excluded from this study if they had a sleep disorder diagnosis before TS. To minimize potential selection bias resulting from the use of unbalanced covariates in observational studies, we employed propensity score matching (PSM) to reduce selection bias due to unbalanced covariates in this observational study. Specifically, an optimized matching mode was used with a caliper width of 0.2 of the pooled standard deviation of the logit of the propensity score, matching each TS patient to four controls in a 1:4 ratio. To assess covariate balance, standardized mean differences (SMD) were calculated, with a threshold of SMD < 0.1 indicating adequate balance. PSM is a statistical matching technique that can be used to reduce potential confounding caused by unbalanced covariates in nonexperimental settings 10, 11. A propensity score is a probability calculated using a logistic regression model. The score is a unit of a certain characteristic assigned to a patient with TS. These scores can reduce or eliminate selection bias in observational studies by accounting for the characteristics of controls. The variables used for matching were sex, age, insured salary, urbanization level, Charlson comorbidity index (CCI), and year of inclusion in the study. The CCI was used to estimate comorbidity severity in this study. The CCI classifies comorbidities into 17 types and converts them into weighted scores; the scores are then summed to calculate the corresponding CCI scores 12. After matching, this study comprised 13 646 patients with TS and 54 584 matched controls from the general population as the comparison cohort. The patient selection process is presented in **Figure 1**.

### Study design

This retrospective cohort study investigated the risk of sleep disorders (ICD-9-CM code 307.4; ICD-10-CM codes G47.8 and G47.9) among patients with TS. The comorbidities included in the analysis were ADHD (ICD-9-CM code 314.0), OCD, (ICD-9-CM code 300.3), anxiety (ICD-9-CM code 300.0), depression (ICD-9-CM codes 296.2 and 296.3), obesity (ICD-9-CM code 287.0), atopic dermatitis (ICD-9-CM code 691), allergic rhinitis (ICD-9-CM code 477), and chronic urticarial (ICD-9-CM code 708). The date of TS diagnosis was the observation start date for patients with TS, and after matching, the same date was assigned as the observation start date for controls. All patients were followed up from the observation start date until death, diagnosis of sleep disorders, or the end date of 2018.

### Statistical analysis

All statistical analyses were conducted using SAS software version 9.4 (SAS Institute, Cary, NC, USA), and statistical significance was indicated by *p* < 0.05. Chi-square tests and SMD were used to evaluate the distributions of baseline characteristics. A Cox proportional hazards model was employed to investigate the association between TS and sleep disorders, adjusting for the following relevant variables: sex, age, insured salary, urbanization level, CCI, ADHD, OCD, anxiety, depression, obesity, atopic dermatitis, allergic rhinitis, and chronic urticaria. The results are presented as hazard ratios (HRs) with 95% confidence intervals (CIs). Besides, we used the variance inflation factor (VIF) to assess multicollinearity among variables and confirmed that all VIF of variables were below 10, suggesting no substantial multicollinearity in the model. Sensitivity analyses were conducted to examine the risk of sleep disorders among patients with TS at <1-, 1-5-, and >5-year follow-up periods.

## Results

**Table 1** provides the baseline characteristics of patients with TS and matched controls. Of the 68 230 study individuals, 56 620 were male patients, and 11 610 were female patients. Of these patients, 13 646 patients had TS, and 54 584 were from the general population. The average ages of patients with TS and controls were 11.32 ± 7.82 and 14.29 ± 11.91 years, respectively. Sex, age, insured salary, urbanization, and CCI did not differ significantly between the groups (*p* > 0.05). Among patients with TS, 2 260 (16.56%) had ADHD, 188 (1.38%) had OCD, 526 (3.85%) had anxiety, 79 (0.58%) had depression, 437 (3.20%) had atopic dermatitis, 4 324 (31.69%) had allergic rhinitis, and 525 (3.85%) had chronic urticarial. Post-matching results confirmed that all SMD values were below 0.1 and p-values exceeded 0.05, indicating no significant differences between the TS and control groups on each matching variable.

**Table 2** presents the incidence rate of sleep disorders in patients with TS and controls. In total, 1 706 patients (2.50%) developed sleep disorders, of whom 1 158 (4.02%) had TS. The incidence rates of sleep disorders were 4.61 and 2.40 per 1 000 person-years in patients with TS and controls, respectively. In total, 406 female (3.50%) and 1 300 male (2.30%) patients developed sleep disorders; the incidence rates were 3.95 and 2.61 per 1 000 person-years, respectively. Patients with ADHD, OCD, anxiety, or depression were more likely to develop sleep disorders than were those without comorbidities.

**Table 3** presents the risk of incident sleep disorder of patients with TS and the controls for comparison. With confounding variables adjusted for, the analysis revealed that patients with TS had a significantly higher risk of incident sleep disorders (adjusted HR [aHR] = 1.76, 95% CI = 1.58-1.96) than controls. Male patients had a lower risk of incident sleep disorders (aHR = 0.68, 95% CI = 0.61-0.76) than female patients. The risk of incident sleep disorders increased with age or CCI. Regarding comorbidities, patients with ADHD (aHR = 1.35, 95% CI = 1.09-1.67) or anxiety (aHR = 2.33, 95% CI = 1.88-2.88) had a high risk of incident sleep disorders after adjustment for relevant variables.

Furthermore, we estimated the risk of sleep disorders in different follow-up periods (Table 4). Compared with controls, patients with TS had the highest risk (aHR = 3.68, 95% CI = 2.62-5.17) of incident sleep disorders in <1 year. At the 1-5-year follow-up (aHR = 1.79, 95% CI = 1.47-2.17) and >5-year follow-up (aHR = 1.52, 95% CI = 1.31-1.76), patients with TS still had a high risk of sleep disorders.

## Discussion

In this large-scale, nationwide, population-based cohort study, patients with TS had a higher risk of sleep disorders than controls from the general population. Our study confirms that TS is a risk factor for sleep disorders. We also found that patients comorbid with ADHD, anxiety, or depression had a high risk of sleep disturbance. The findings suggest that neuropsychiatric comorbidities increase the likelihood of sleep disorders in patients with TS. In our cohort, the risk of sleep disorders was significantly higher in patients with TS aged more than 18 years and 7-18 years than in those aged less than 7 years. The risk of sleep disorders was the highest in patients diagnosed with TS within less than 1 year.

In clinical settings, approximately half of the children and adolescents with TD and more than 80% of patients with TS have at least one comorbid developmental neuropsychiatric disorder, and approximately 60% of patients with TS have two or more comorbidities (ADHD, OCD, depression, anxiety, and sleep disorders) 1, 4, 13. The most common and well characterized of these neuropsychiatric comorbidities are ADHD and OCD, with prevalence rates of 50% to 60% and of 36% to 50%, respectively 3, 14-16.

Several studies have indicated that sleep disorders and sleep problems are frequent in patients with TS, with a prevalence rate of 12% to 62% 17-19. TD is associated with high rates of neuropsychiatric comorbidities, particularly ADHD and OCD 20. Sleep disturbance, including difficulties with sleep initiation and maintenance, is common in children with TD, with rates as high as 65% in TD patients without neuropsychiatric comorbidities 21. Although the cause of frequent sleep disturbances in patients with TS remains uncertain, several general theories have been proposed. A study indicated that tic behaviors may occur in various stages of sleep, contributing to overall increased movement during both rapid eye movement (REM) and non-REM sleep and therefore affecting normal sleep patterns 6. Both dopamine (DA) and serotonin (5-HT) systems and their interaction are involved in the regulation of the circadian rhythm 22, 23. Furthermore, decreased serotonin levels lead to increased phasic DA release as well as alterations in the expression of DA D2 receptor (D2R) and serotonin receptor 2A (5-HT2AR) in TS 24, 25. D2R 26 and 5-HT2AR 27 are implicated in the physiological regulation of the circadian rhythm.

In our cohort study, patients with TS had a higher risk of sleep disorders than controls. The odds were 1.7 times higher in patients with TS than in controls from the general population. Increasing evidence has suggested that sleep disturbances are more common in children with TD 28, 29; it is especially frequent in patients with severe and chronic symptoms 30. Children with TD have a shorter sleep duration than children without TD, and sleep disturbances worsen with age 31. Children with TD are at higher risk of sleep disturbances, and they have a higher prevalence of some sleep disorders, such as sleep-related movement disorders 31. A previous study reported that the prevalence of sleep problems in patients with TS was approximately 60% 17. A recent study also indicated that sleep disorders occur in 64% of patients with TS 32. A systematic review revealed a high prevalence of 9.7% to 80.4% for sleep difficulties in children with TS 29. Approximately one-third of patients with TS have insomnia. Compared with the general population, the period prevalence of insomnia translates into a 6.7-fold increased likelihood in the TS group 33.

In the present study, the risk of sleep disorders was significantly higher in patients aged more than 18 years (*7*- to 8-fold) and 7-18 years (*2*- to 3-fold) than in those aged less than 7 years. In addition, female patients were at higher risk of sleep disorders than male patients. Our study also revealed that patients who had higher CCI scores were at higher risk, especially those with CCI scores of more than 2. A study demonstrated that older adolescents had lower sleep quality than younger children 31. Consistently, our study result revealed that patients aged more than 18 years were at higher risk of sleep disorders than patients aged less than 7 years. The underlying mechanisms of sleep disturbances in children with TS are not fully understood, though several possible explanations have been proposed. Previous study indicated that a dysfunction in the dopaminergic system, likely involving increased dopaminergic activity, as indicated by symptom improvement with dopamine-depleting medications (such as tetrabenazine) 34. It has been suggested that environmental influences on genetic predisposition may have an epigenetic basis. Findings from magnetic resonance spectroscopy and positron emission tomography studies indicate the involvement of other neurotransmitters, such as GABA and glutamate 35. There is substantial evidence indicating the involvement of the cortical-basal ganglia-thalamocortical circuits, which play a key role in movement regulation and control 31. Several neurotransmitters, particularly acetylcholine, norepinephrine, serotonin, histamine, and GABA, play a crucial role in regulating sleep and wakefulness, supporting a pathological basis for the heightened sleep disturbances observed in TS 31,36.

In this cohort study, patients with neuropsychiatric comorbidities exhibited an increased likelihood of sleep problems**.** Hence, we also investigated neuropsychiatric comorbidities as risk factors for and the risk of incident sleep disorder. The results revealed that patients comorbid with ADHD or anxiety had a higher risk for sleep disturbance. Most patients with TS have comorbid conditions, including neuropsychiatric disorders, which are more impairing than the tics themselves 37, 38. A population-based case-control study revealed that TS is a risk factor for sleep disorders, and children with TS having comorbidities had an increased risk of sleep disorders 39. A study indicated that insufficient sleep in young TD patients persists independently of comorbidity or psychiatric drug status 21. Previous studies have also demonstrated sleep deficits in young TD patients without comorbid conditions 32. Strong evidence suggests that sleep is negatively affected in patients with TD, particularly children with TD; a direct association between daytime tic severity and sleep disturbance has also been suggested 6. Children with TDs had a higher frequency of sleep disorders than controls 19, 40. The most severe tics were found in patients with both ADHD and OCD 41 42. Children with TS often present with several comorbidities. ADHD is the most common comorbidity 32. A relationship of TD with ADHD has been found; ADHD has negative effects on sleep in terms of several indicators 31. Previous studies have indicated that youth with TD and comorbid ADHD reported higher sleep disturbance relative to patients with TD without psychiatric comorbidity 32, 43. Another study revealed that children with TS with or without comorbid ADHD exhibit sleep disorders 32. A meta-analysis of actigraphy-derived parameters revealed that longer sleep onset latency and poorer sleep efficiency are the most common findings in patients with TS with or without comorbid ADHD 44. However, patients with TS with comorbid ADHD and/or OCD have an increased likelihood of sleep problems 41, 43, 45; this finding suggests that sleep disorders in patients with TS are attributed to comorbidities rather than TS itself. Several studies have shown that anxiety is a common comorbidity in patients with TS, with an incidence ranging from 11% to 70.3% 15, 46. Patients with chronic TDs and anxiety disorders reported higher frequency of sleep-related problems than those without comorbid anxiety disorder 47. The impact of ADHD, other comorbidities (such as anxiety), and medication effects on sleep disturbances in individuals with TS has been recognized 21,32,34,39. A study found that neither medications nor comorbidities appeared to predict sufficient sleep duration in children with TS, suggesting that sleep disturbances are an inherent feature of TS rather than solely a consequence of comorbid conditions or medication effects 21. A population-based study in Taiwan found that the prevalence of Tourette syndrome and chronic tic disorders was highest (57.13%) in patients younger than 10 years old, followed by a lower prevalence (23.53%) in those aged 10-19 years 48. Another study from Taiwan reported that the prevalence of OCD peaked in males between 18-24 years and in females between 35-44 years, with the next highest prevalence occurring in males aged 25-34 years and females aged 25-34 years 49. The peak prevalence ages for Tourette syndrome and OCD differed. Therefore, we included patients with Tourette syndrome comorbid OCD, which had a relatively low prevalence, potentially affecting the observed lack of association with sleep disorders compared to the control group.

In our cohort study, the risk of sleep disorders was the highest in patients diagnosed with TS within less than 1 year, with an aHR of 3.81, and the aHRs for sleep disorders were 1.84 and 1.50 at 1-5-and >5-year follow-up, respectively. Moreover, the risk of sleep disorders in patients with TS was identified most commonly in the earlier follow-up period. Our study result is similar to that of a population-based case-control study 39. On average, the tics in patients with TS increase in severity and peak at the age of 10-12 years; they then tend to improve gradually during adolescence; however, symptom progression substantially differs across patients 3, 50. Over the years, tic severity typically increases and reaches a peak between 8 and 12 years of age. Tic severity usually peaks during the second decade of life, and in the majority of children, tic severity improves by the late teens and early adulthood 16. By the end of the second decade of life, many patients are mostly tic-free 51. However, approximately 20% of patients with TS continue to experience clinically impairing tics into adulthood.

Our study has several strengths. In our study, patients were selected from the total population of a nationwide cohort in Taiwan; thus, the large sample size is representative, increasing statistical precision. We believe that the combination of the NHIRD with multiple data sources would be useful. The population-based design also minimized selection bias, which is common in observational studies. Although a prospective population-based cohort study is ideal for exploring risk factors, a retrospective population-based cohort study using insurance data is a suitable alternative.

This study has several limitations. First, due to the nature of the National Health Insurance Research Database (NHIRD), which relies on claims data coded with ICD-9-CM and ICD-10-CM for sleep disorders, we were unable to differentiate specific sleep problems such as insomnia, excessive daytime somnolence, restless legs syndrome, enuresis, or REM sleep behavior disorder. The NHIRD does not provide detailed clinical information, such as symptom-specific assessments, sleep logs, or polysomnography results, which limits our ability to analyze the prevalence and impact of individual sleep disorders among TS patients. Future studies incorporating clinical evaluations or specialized sleep assessment tools could further elucidate the specific sleep disturbances associated with TS. Second, surveillance bias was unavoidable in this population-based cohort study, where data had been gathered in clinical settings. Children with TS are more likely to be diagnosed by *pediatric neurologists* or child *psychiatrists*, and the parents of TS patients may pay more attention to comorbidities, including sleep disorders and neuropsychiatric disorders.

## Conclusion

TS is a risk factor for sleep disorders. Our study also indicated that patients with comorbid ADHD or anxiety had a higher risk of sleep disturbance, suggesting that the increased risk of sleep disorders is associated with comorbidities. In this cohort, the risk of sleep disorders was significantly higher in patients with TS aged more than 18 years than in those aged less than 7 years, and the risk of sleep disorders was the highest in patients diagnosed with TS within less than 1 year.

## Figures and Tables

**Table 1:** Baseline characteristics of study subjects after matching.

Variables	Total		General patients		Tourette syndrome			p-value	SMD ^3^
	N	%	N	%	N		%		
Total	68,230	100.00	54,584	100.00	13,646		100.00		
Sex ^1^								1.000	0
Female	11,610	17.02	9,288	17.02	2,322		17.02		
Male	56,620	82.98	45,296	82.98	11,324		82.98		
Age (year) ^1^								0.998	0.003
<7	11,560	16.94	9,246	16.94	2,314		16.96		
7-18	49,952	73.21	39,962	73.21	9,990		73.21		
>18	6,718	9.85	5,376	9.85	1,342		9.83		
Mean ± SD	13.69 ± 11.27		14.29 ± 11.91		11.32 ± 7.82				
Insured salary (NTD) ^1,2^								0.998	0.003
≤19,200	21,863	32.04	17,487	32.04	4,376		32.07		
19,201-21,900	14,075	20.63	11,254	20.62	2,821		20.67		
21,900-36,300	15,155	22.21	12,128	22.22	3,027		22.18		
≥36,301	17,137	25.12	13,715	25.13	3,422		25.08		
Urbanization ^1,2^								1.000	0
Level 1	21,896	32.09	17,515	32.09	4,381		32.10		
Level 2	21,405	31.37	17,120	31.36	4,285		31.40		
Level 3	11,656	17.08	9,324	17.08	2,332		17.09		
Level 4	8,272	12.12	6,616	12.12	1,656		12.14		
Level 5	947	1.39	758	1.39	189		1.39		
Level 6	1,813	2.66	1,459	2.67	354		2.59		
Level 7	2,241	3.28	1,792	3.28	449		3.29		
CCI score ^1,3^								0.998	0.002
0	47,384	69.45	37,904	69.44	9,480		69.47		
1	19,353	28.36	15,485	28.37	3,868		28.35		
≥2	1,493	2.19	1,195	2.19	298		2.18		
ADHD ^3^								<0.001	0.069
No	64,872	95.08	53,486	97.99	11,386		83.44		
Yes	3,358	4.92	1,098	2.01	2,260		16.56		
OCD ^3^								<0.001	0.073
No	68,009	99.68	54,551	99.94	13,458		98.62		
Yes	221	0.32	33	0.06	188		1.38		
Anxiety								<0.001	0.104
No	67,314	98.66	54,194	99.29	13,120		96.15		
Yes	916	1.34	390	0.71	526		3.85		
Depression								<0.001	0.533
No	68,113	99.83	54,546	99.93	13,567		99.42		
Yes	117	0.17	38	0.07	79		0.58		
Obesity								0.711	0.070
No	68,113	99.83	54,492	99.83	13,621		99.82		
Yes	117	0.17	92	0.17	25		0.18		
Atopic dermatitis								0.009	0.034
No	66,273	97.13	53,064	97.22	13,209		96.80		
Yes	1,957	2.87	1,520	2.78	437		3.20		
Allergic rhinitis								<0.001	0.848
No	55,542	81.40	46,220	84.68	9,322		68.31		
Yes	12,688	18.60	8,364	15.32	4,324		31.69		
Chronic urticarial								<0.001	0.221
No	66,039	96.79	52,918	96.95	13,121		96.15		
Yes	2,191	3.21	1,666	3.05	525		3.85		
									

^1^ Variables for propensity score matching. ^2^ NTD is New Taiwan Dollar (NTD 1 ≈ USD 0.034); Urbanization Level 1 denoted the highest degree of urbanization, whereas level 7 denoted the lowest degree of urbanization.^3^ Abbreviations: CCI, Charlson comorbidity index; ADHD, attention deficit hyperactivity disorder; OCD, obsessive-compulsive disorder. SMD; standardized mean differences.

**Table 2:** The incident rate of sleep disorder.

Variables	Sleep disorder					
	No		Yes		IR ^1^	p-value
	N	%	N	%		
Total	66,524	97.50	1,706	2.50	2.84	
Patient group						<0.001
General	53,426	97.88	1,158	2.12	2.40	
Tourette syndrome	13,098	95.98	548	4.02	4.61	
Sex						<0.001
Female	11,204	96.50	406	3.50	3.95	
Male	55,320	97.70	1,300	2.30	2.61	
Age (year)						<0.001
<7	11,438	98.94	122	1.06	1.17	
7-18	48,897	97.89	1,055	2.11	2.39	
>18	6,189	92.13	529	7.87	9.43	
Mean ± SD	13.49 ± 11.00		21.64 ± 17.35			
Insured salary (NTD) ^2^						<0.001
≤19,200	21,126	96.63	737	3.37	3.33	
19,201-21,900	13,797	98.02	278	1.98	2.47	
21,900-36,300	14,831	97.86	324	2.14	2.79	
≥36,301	16,770	97.86	367	2.14	2.43	
Urbanization ^2^						0.051
Level 1	21,285	97.21	611	2.79	3.08	
Level 2	20,894	97.61	511	2.39	2.74	
Level 3	11,390	97.72	266	2.28	2.64	
Level 4	8,068	97.53	204	2.47	2.90	
Level 5	926	97.78	21	2.22	2.56	
Level 6	1,775	97.90	38	2.10	2.17	
Level 7	2,186	97.55	55	2.45	2.80	
CCI score ^3^						<0.001
0	46,324	97.76	1,060	2.24	2.54	
1	18,835	97.32	518	2.68	3.06	
≥2	1,365	91.43	128	8.57	9.30	
ADHD ^3^						0.003
No	63,276	97.54	1,596	2.46	2.77	
Yes	3,248	96.72	110	3.28	4.24	
OCD ^3^						<0.001
No	66,322	97.52	1,687	2.48	2.82	
Yes	202	91.40	19	8.60	9.57	
Anxiety						<0.001
No	65,713	97.62	1,601	2.38	2.69	
Yes	811	88.54	105	11.46	14.70	
Depression						<0.001
No	66,431	97.53	1,682	2.47	2.80	
Yes	93	79.49	24	20.51	22.71	
Obesity						
No	66,412	97.50	1,701	2.50	2.83	0.219
Yes	112	95.73	5	4.27	5.39	
Atopic dermatitis						0.080
No	64,604	97.48	1,669	2.52	2.85	
Yes	1,920	98.11	37	1.89	2.23	
Allergic rhinitis						0.694
No	54,147	97.49	1,395	2.51	2.82	
Yes	12,377	97.55	311	2.45	2.89	
Chronic urticarial						0.066
No	64,401	97.52	1,638	2.48	2.81	
Yes	2,123	96.90	68	3.10	3.62	

^1^ IR, incidence rate per 1,000 person-year. ^2^ NTD is New Taiwan Dollar (NTD 1 ≈ USD 0.034); Urbanization Level 1 denoted the highest degree of urbanization, whereas level 7 denoted the lowest degree of urbanization. ^3^ Abbreviations: CCI, Charlson comorbidity index; ADHD, attention deficit hyperactivity disorder; OCD, obsessive-compulsive disorder.

**Table 3:** The risk of sleep disorder between Tourette syndrome and general patients.

Variables	Unadjusted model					Adjusted model				
	HR	95% CI			p-value	HR	95% CI			p-value
Patient group										
General (ref.)	1					1				
Tourette syndrome	1.93	1.74	-	2.13	<0.001	1.76	1.58	-	1.96	<0.001
Sex										
Female (ref.)	1					1				
Male	0.66	0.59	-	0.74	<0.001	0.68	0.61	-	0.76	<0.001
Age (year)										
<7 (ref.)	1					1				
7-18	2.08	1.72	-	2.51	<0.001	2.18	1.80	-	2.64	<0.001
>18	8.23	6.76	-	10.02	<0.001	7.76	6.32	-	9.53	<0.001
Insured salary (NTD) ^1^										
≤19,200 (ref.)	1					1				
19,201-21,900	0.83	0.72	-	0.96	0.010	0.99	0.86	-	1.14	0.864
21,900-36,300	0.91	0.80	-	1.04	0.171	0.97	0.85	-	1.11	0.652
≥36,301	0.77	0.68	-	0.87	<0.001	0.84	0.74	-	0.96	0.009
Urbanization ^1^										
Level 1 (ref.)	1					1				
Level 2	0.91	0.81	-	1.02	0.099	0.88	0.78	-	0.99	0.034
Level 3	0.88	0.76	-	1.01	0.072	0.91	0.78	-	1.05	0.176
Level 4	0.96	0.82	-	1.13	0.646	0.97	0.83	-	1.14	0.741
Level 5	0.85	0.55	-	1.31	0.459	0.79	0.51	-	1.22	0.282
Level 6	0.69	0.50	-	0.96	0.028	0.68	0.49	-	0.95	0.023
Level 7	0.93	0.70	-	1.22	0.592	0.91	0.69	-	1.20	0.512
CCI score ^2^										
0 (ref.)	1					1				
1	1.21	1.09	-	1.34	<0.001	1.28	1.15	-	1.43	<0.001
≥2	3.57	2.97	-	4.28	<0.001	1.78	1.47	-	2.17	<0.001
ADHD ^2^										
No (ref.)	1					1				
Yes	1.63	1.35	-	1.98	<0.001	1.35	1.09	-	1.67	0.005
OCD ^2^										
No (ref.)	1					1				
Yes	3.34	2.12	-	5.25	<0.001	0.96	0.60	-	1.55	0.875
Anxiety										
No (ref.)	1					1				
Yes	5.67	4.65	-	6.90	<0.001	2.33	1.88	-	2.88	<0.001
Depression										
No (ref.)	1					1				
Yes	7.85	5.25	-	11.75	<0.001	1.49	0.97	-	2.29	0.069
Obesity										
No (ref.)	1					1				
Yes	1.97	0.82	-	4.74	0.130	1.57	0.65	-	3.78	0.315
Atopic dermatitis										
No (ref.)	1					1				
Yes	0.79	0.57	-	1.10	0.164	0.94	0.67	-	1.31	0.709
Allergic rhinitis										
No (ref.)	1					1				
Yes	1.04	0.92	-	1.18	0.488	1.08	0.94	-	1.24	0.260
Chronic urticarial										
No (ref.)	1					1				
Yes	1.30	1.02	-	1.66	0.034	1.24	0.97	-	1.58	0.083

^1^ NTD is New Taiwan Dollar (NTD 1 ≈ USD 0.034); Urbanization Level 1 denoted the highest degree of urbanization, whereas level 7 denoted the lowest degree of urbanization. ^2^ Abbreviations: CCI, Charlson comorbidity index; ADHD, attention deficit hyperactivity disorder; OCD, obsessive-compulsive disorder.

**Table 4:** Sensitivity analysis to investigate the risk of sleep disordor at different follow-up periods.

Variables	General patients		Tourette syndrome		Adjusted model ^1^				
	Events	IR	Events	IR	HR	95% CI			p-value
Follow-up year									
<1 year	72	1.32	82	6.03	3.68	2.62	-	5.17	<0.001
1-5 year	353	1.30	178	2.62	1.79	1.47	-	2.17	<0.001
>5 year	733	1.52	288	2.42	1.52	1.31	-	1.76	<0.001

^1^ All models were analyzed using the Cox proportional hazards model. Extraneous factors adjusted in the model were sex, age, insured salary, urbanization, CCI, and comorbidities.
